# Prediction of cost and schedule performance in post-hurricane reconstruction of transportation infrastructure

**DOI:** 10.1371/journal.pone.0282231

**Published:** 2023-03-29

**Authors:** Elnaz Safapour, Sharareh Kermanshachi, Behzad Rouhanizadeh

**Affiliations:** 1 Department of Civil and Environmental Engineering, University of New Orleans, New Orleans, Louisiana, United States of America; 2 Department of Civil Engineering, University of Texas at Arlington, Arlington, Texas, United States of America; 3 Department of Civil, Construction, and Environmental Engineering, University of Alabama, Tuscaloosa, Alabama, United States of America; Shahrood University of Technology, ISLAMIC REPUBLIC OF IRAN

## Abstract

This study aimed to develop predictive models that could be used to estimate the cost and schedule performance of reconstruction of transportation infrastructure damaged by hurricanes and to determine the predictors that are robustly connected to the developed models. Stepwise multiple linear regression and extreme bound analysis (EBA) were used to develop the models and determine the robust and fragile predictors, respectively. The results demonstrated that seven cost performance predictors and nine schedule performance predictors accounted for Adjusted *R*-Squared of 92.4% and 99.2%, respectively. The results of the EBA revealed that four cost and seven performance predictors were robustly connected to the developed cost and schedule performance predictive models. It was concluded that increases in laborers’ wages, the number of inspections, information and data management, and addressing safety and environmental issues prior to a project’s execution were predictors of both the cost and schedule performance of reconstruction projects. The outcomes of this study provide knowledge and information that will be helpful to decision-makers who are responsible for mitigating delays and cost overruns, and effectively allocating their limited resources available following a disaster.

## Introduction

The recovery process of returning a community to its pre-hurricane condition needs to begin soon after a disaster occurs. Damaged transportation systems need to be restored as quickly as possible to mitigate socioeconomic disruptions [1], enable the rapid response of emergency management teams, and facilitate optimal traffic flow so that the supply chain of essential products and resources is unimpeded [2]. The rehabilitation of transportation infrastructure is often costly [3–5], and a lack of sufficient financial resources lessens the chance of the projects being accomplished on time and within the baseline budget [6]. Local governments are often under pressure from the public when contractors cannot complete the projects on time, and decision-makers and project managers are tasked with prioritizing needs and effectively allocating the limited available resources.

Furthermore, equipment, materials, and labor are usually in short supply following disasters, particularly hurricanes, and prices are often inflated [7, 8], all of which lead to time delays, cost escalations, and decreased success of some projects. Risks and uncertainties arise from the chaos and chaotic condition following the hurricane and merit further study [4, 9, 10]. Although a few studies have been conducted to identify the root causes of cost escalations and delays pertaining to large-scale reconstruction projects, the current literature lacks applicable models to predict cost overruns and time delays. In addition, rarely studies have been conducted to help decision-makers and stakeholders prioritize resources to allocate them effectively through the reconstruction of transportation infrastructure, as there are limited available resources following disasters, particularly hurricanes. Therefore, the aims of this study were (1) to develop models that can predict the cost and schedule performance of reconstructing transport infrastructure damaged by hurricanes; and (2) to determine which of the predictors are robustly connected to the developed models. Four objectives were formulated to achieve these aims: (1) investigate the predictors that contribute to the success of the reconstruction projects, (2) generate a model that can predict the cost performance of reconstruction projects, (3) generate a model that can predict the schedule performance of reconstruction projects, and (4) identify which predictors are robust and which are fragile. The developed two predictive models and determined robust predictors based on projects’ conditions aftermath of the hurricanes help project managers and decision-makers make effective plans prior to reconstructing the transportation infrastructure to reduce uncertainties and minimize the consequences on the projects’ cost and schedule performances and enhance projects’ productivity.

## Literature review

### Hurricanes

Hurricanes are one of the most destructive types of natural disasters in the United States, as they levy many disruptions, significant financial losses, and extensive damage to the infrastructure [11, 12]. Hurricane Andrew led to $26.5 billion in damages in Florida in 1992 [13], and Hurricanes Rita and Katrina caused significant socioeconomic losses and damages, with total dollar values considerably higher than what was estimated. The total indirect and direct cost of damages from Hurricane Katrina was roughly $1,900 billion, while it was estimated to be $160 billion [14]. Similarly, the total cost of losses and damages from Hurricane Rita was approximately $120 billion, while it was estimated to be $10 billion [15].

### Reconstruction of transportation infrastructure

The transportation sector’s losses and damages are among the largest after a natural disaster, and reconstruction is expensive [3]. For instance, after a natural disaster occurred in Sri Lanka, the damage to the transportation sector amounted to 22% of the total losses that occurred by the disaster. Similarly, the reconstruction of the substantial damages sustained by bridges following Hurricane Katrina in Louisiana (2005), amounted to more than $1 billion [16]. The reconstruction process of transportation infrastructure needs to begin as soon after the destructive event as possible, as the time necessary to complete it is often longer than was predicted. Many researchers believe that cost escalations are one of the most critical issues encountered in reconstruction, as they commonly need significant financial support and take longer to accomplish than anticipated [17].

### Criteria for success

The existing literature defines a successful construction project by a number of criteria. Multiple researchers and practitioners state that when a project is completed on time and meets all of the clients’ objectives and requirements, it can be considered successful [18, 19]. Jha and Iyer [20] concluded that time, cost, quality, coordination and management, commitment, and competence are necessary to the success of a project. Multiple studies have been performed on construction projects that were deemed successful by the mentioned criteria [18, 19].

Many researchers and practitioners believe that time delays are one of project managers’ primary challenges [21, 22]. Ahmed et al. (2002) [21] stated that time delays are a common problem in construction projects, and Thomsen et al. (2010) [23] estimated that about half of U.S. construction projects experience considerable time delays.

Cost escalations in construction and reconstruction projects happen when the actual costs exceed the baseline budget. Multiple researchers and practitioners consider them a common phenomenon that seriously decreases the chance of a project’s success [24]. Flyvbjerg et al. [25] concluded that roughly 90% of all construction and reconstruction projects experience cost escalations. Similarly, Shane et al. (2009) [26] stated that approximately half of U.S. large-scale construction projects experience cost escalations. The negative effects of increased construction costs are not solely financial; however, they can also impact other aspects of a project, such as its quality and safety [24, 27].

### Causes of reconstruction failure

The determinants for success or failure are unique to each post-hurricane reconstruction project. The project’s uniqueness, environmental and safety issues, the decision-makers attitudes, and various other factors can play major or minor roles in its success. Many studies have been performed to determine critical causes of failure in reconstruction projects [8, 28, 29], some of which are presented in Table 1.

**Table 1:** Main causes of reconstruction failures after disasters.

Key Causes	Previous Study
Delay in delivering resources	[29]
Finance and limitation of funds	[6, 7]
Inappropriate assessment	[30]
Communication and coordination	[8]
Ineffective design	[31]
Lack of appropriate transportation	[32]
Temporary paths	[33]
Inadequacy of resource procurement	[8]
Difficulties in damage evaluation	[28]
Unavailability of human resources	[34]
Unavailability of material resources	[34, 35]
Low pace of decision-making	[36, 37]
Number and Quality of Inspection	[38]
Engineering mobilization	[38]
Inability in relocation of functions	[6]
Inflation	[8]
Permitting and consenting	[8]

### Knowledge gap

Various challenges, risks, and uncertainties that increase the cost and duration of reconstructing transportation infrastructure damaged by hurricanes are attributed to the dynamic and chaotic nature of the post-hurricane condition. The literature lacks sufficient studies investigating the impacts of these challenges, risks, and uncertainties on the cost and schedule performance of reconstruction projects. Therefore, this study aimed to fill the stated gap of knowledge by minimizing cost overruns and schedule delays in reconstruction of transportation infrastructure damaged by hurricanes. To this aim, two models were developed to predict the cost and schedule performance of the post-hurricane reconstruction of transportation infrastructure projects. In addition, this study determined which predictors were robustly connected to the developed equations. The outcomes will help project managers prioritize the resources usually limited following hurricanes to allocate them to reconstruction projects effectively.

## Research methodology

### Overview of the study procedure

**Fig 1:**
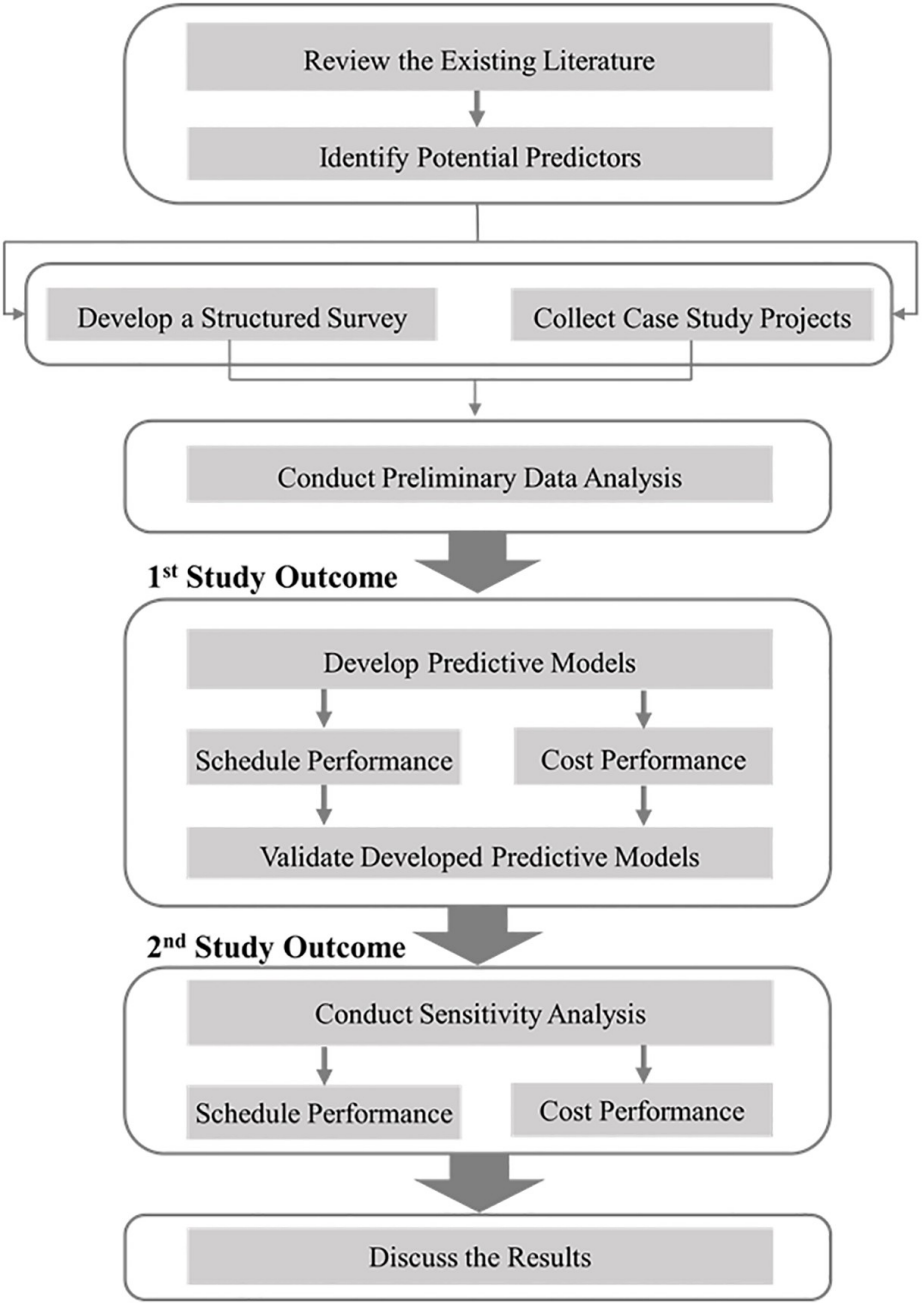
Overview of the study procedure.

The primary purpose of this study was to develop two models to predict cost and schedule performance in the post-hurricane reconstruction of transportation infrastructure. Fig 1 depicts the overview of the study procedure. A comprehensive literature review was conducted to investigate the factors causing the reconstruction failure and identify the potential predictors that potentially affect the cost and schedule performance of reconstructing damaged transportation infrastructure. Then, a research team provided a list of decision-makers, including directors and project managers, involved in reconstructing transportation infrastructures damaged by hurricanes. The research team contacted and asked these decision-makers to share the data and information from previous projects. The information and data were gathered from a set of completed reconstruction projects of transportation infrastructure affected by hurricanes. Since data from some aspects of collected case studies was missed, a structured survey was developed, and the research team contacted the same decision-makers and asked them to complete the survey associated with the same case studies; so, the same number of the survey was collected. Next, data was analyzed, and the method of stepwise multiple linear regression was implemented to generate two models, one to predict the cost performance and one to predict the schedule performance. The method of residual analysis was implemented to evaluate the performance of the statistical model, and a sensitivity analysis was conducted using the Extreme Bound Analysis (EBA) method to identify the level of connection between the predictors and the corresponding predictive model.

### Definition of the predicted values

As this study aimed to predict the project cost and schedule performance, these need to be defined. The two measures of cost and schedule performances of the reconstruction projects were calculated using collected data and information. These measures of cost and schedule performances are presented as follows:

$$\text{Cost}\;\text{performance}=\left[\left(\text{actual}\;\text{cost}-\text{baseline}\;\text{budget}\right)÷\text{baseline}\;\text{budget}\right]\times 100$$ (1)

$$\text{Schedule}\;\text{performance}=\left[\left(\text{actual}\;\text{duration}-\text{baseline}\;\text{schedule}\right)÷\text{baseline}\;\text{schedule}\right]\times 100$$ (2)

It must be mentioned that the cost performance and schedule performance are expressed as categories as the following: (1) cost performance above 10% is labeled poor cost performance, and cost performance less than 10% is labeled a good cost performance; and (2) poor schedule performance is considered more than 20%, and schedule performance less than 20% is considered a good schedule performance [39].

### List of potential predictors

The existing literature was comprehensively reviewed in detail to identify the potential cost and schedule performance predictors in the post-hurricane reconstruction of transportation infrastructure. Accordingly, the literature review for this study encompassed 209 relevant journal articles, conference proceedings, theses and dissertations, and project reports, approximately 75% of which were peer-reviewed journal articles because of the rigorous review process. The following criteria were developed for those that would be included in the database:

1. The articles should have been published over the last 22 years,
2. The articles should have been published by a prestigious publisher, and
3. The articles should be relevant to post-hurricane reconstruction of transportation infrastructure, including highways, roadways, and bridges.

After all of the articles had been screened to evaluate their eligibility and quality, the 89 that remained were reviewed in detail to gather data and information about the potentially influential and critical predictors that affect the cost and schedule performance of post-hurricane reconstruction of transport infrastructure. The research group performed two main steps to identify potential predictors: (1) identifying the predictors that could affect the cost and schedule performance, and (2) determining which of them were most frequently cited, and retaining those, as presented in Table 2.

**Table 2:** List of potential cost and schedule performance predictors and type of survey questions.

Category	List of Potential Predictors	Type of question
Physical Characteristics	P1. Number of main/truck lines	Numerical value
	P2. Total lengths	Numerical value
	P3. Level of complexity	Seven-point Likert scale
	P4. Distance from highly-populated area	Numerical value
Damaging Level	P5. Level of damage	Seven-point Likert scale
	P6. Level of traffic disturbance	Seven-point Likert scale
Resource	P7. Shortage of experts	Seven-point Likert scale
	P8. Shortage of field labors	Seven-point Likert scale
	P9. Productivity level of contractors	Seven-point Likert scale
	P10. Shortage of materials	Seven-point Likert scale
	P11. Shortage of equipment	Seven-point Likert scale
	P12. Inflation of labor wage	Seven-point Likert scale
	P13. Availability level of on-site infrastructure	Binary
	P14. On-site accommodation level for staff (i.e., welfare facilities on reconstruction including sleeping, dining, and living areas, etc.)	Seven-point Likert scale
	P15. Shortage of supplier	Seven-point Likert scale
Quality	P16. Quality issues of materials	Seven-point Likert scale
	P17. Quality issues of equipment	Seven-point Likert scale
Project Management	P18. Frequency level of logistics management issues	
	P19. Quality of on-site inspection	Seven-point Likert scale
	P20. Frequency of on-site inspection	Seven-point Likert scale
	P21. Information management	Seven-point Likert scale
	P22. Pace of decision-making process	Seven-point Likert scale
	P23. Implementation level of risk management	Seven-point Likert scale
	P24. Coordination	Seven-point Likert scale
	P25. Pace of workers’ mobilization	Seven-point Likert scale
Environment & Safety	P26. Volume of debris	Numerical value
	P27. Addressing the environmental/safety issues prior to the project’s execution	Binary and Numerical value*
	P28. Work suspension	Binary and Numerical value*
Legal	P29. Regulatory requirement	Seven-point Likert scale
Local	P30. Availability of required temporary pathways	Binary

* This question in the survey consists of two parts a and b. In the first part of the question (i.e., part a), the survey respondents were asked to provide information whether this issue existed in the focused project or not; so that the response would be binary. In the second part of the question (i.e., part b), the respondents were asked to provide information about how long the mentioned issue lasted in the project to be solved entirely; therefore, the response would be a numerical value.

### Data collection

To provide input for developing models, practitioners and experts who were involved in relative projects were contacted for interviews. These experts were asked to provide input for the models from records of sample projects with known features corresponding to potential predictors and known performance. Also, the practitioners were asked to provide their judgements on the project features without precise measures. Thus, the research team provided a list of more than 200 practitioners, including directors, project managers, policy makers, and engineers who worked for U.S. companies and agencies involved in reconstructing hurricane-damaged transportation infrastructure. The research team contacted them via emails, and after extensive communication, professionals working in 30 companies and agencies shared comprehensive information and data from 30 completed projects with which they were involved. To fill in the knowledge gaps and gather additional research material, the research team designed a structured survey comprised of 46 questions that were divided into two sections. In the first section, the survey respondents were asked to provide information associated with their profession (e.g., position and work experience) and general information on each case study (e.g., baseline budget, baseline schedule, actual cost, and actual time). In the second section of the survey, each potential predictor became one question. Since potential predictors were classified into eight classes, the second section of the survey consisted of eight subsections. Table 2 shows the mentioned eight classes: physical characteristics, damaging level, resource, quality, project management, environment and safety, legal, and local. This classification helps survey respondents understand the nature and concept of each question better. Table 2 shows the mentioned eight classes. The questions were developed to collect three types of responses: Likert scale, binary, and continuous value. Before distributing the survey, it was pilot tested with five experts to confirm the reliability and validity of the questions associated with the identified potential cost and schedule predictors. Next, the research team asked the professionals from the 30 companies that had already provided their projects’ data to complete the survey online and provide complementary information. After three follow-up emails, all 30 completed the survey, and the results are shown in Table 3.

**Table 3:** Respondents’ demographic information.

Years of Experience	Percentage (%)	Role in the Company	Percentage (%)
Less than 10 years	12.5%	Program Manager	8%
Between 10 and 20 years	21%	Director	17%
Between 21 and 30 years	37.5%	Project Manager	30%
More than 30 years	29%	Engineer	45%

### Data preparation

The questions in the survey were designed to collect three types of responses: Likert-scale, continuous value, and binary response. Therefore, three statistical analysis methods were adopted to determine statistically significant cost performance predictors. Accordingly, two-sample t-test was adopted for numerical responses; Chi-square and Kruskal-Wallis methods were implemented for binary and Likert-scale responses, respectively.

*Reliability test*: As stated earlier, a survey was designed to collect comprehensive data and information from necessary aspects of collected 30 completed reconstruction projects. Cronbach’s alpha test was conducted to investigate the degree of internal consistency (i.e., reliability of the scale used) when the source of the data for developing a model is a survey involving multiple Likert questions, as it is in this research [40]. The Cronbach’s alpha test result ranges from zero to one, and a result higher than 0.7 indicates an adequate degree of internal consistency.

*Z-transformation method to normalize the data*: As the survey was designed to collect three different types of responses (Likert-scale, numerical value, and binary), it is necessary to normalize and standardize the collected data to develop predictive cost and schedule performance models; so, the z-transformation method was implemented to standardize and normalize the data associated with the predictor variables. The normalizing data allows the research team to use different types of data without considering the original scales in developing the predictive cost and schedule performance models. Since part of the data was collected in binary (yes and no), the values were “1” for “yes” and “0” for “no” responses. The z-transformation equation is presented as follows:

$$Z=\frac{\text{x}-\mu }{\sigma }$$ (3)

where x refers to an observation, μ refers to the mean of the distribution, and σ refers to the standard deviation.

### Stepwise multiple linear regression method

The Stepwise multiple linear regression method was implemented to develop two models to predict cost and schedule performance in post-hurricane reconstruction of transportation infrastructure. The Stepwise multiple linear regression method is considered an effective tool for authors and researchers [41] to investigate relationships between influential factors and dependent variables. Its purpose is to develop and formulate an equation to predict the dependent variables by combining the influential variables [42]. Independent variables are entered into the regression equation one at a time. The procedure will continue with the independent variable that contributes the most to the developed and formulated equation according to the value of multiple correlations, R, until adding another independent variable will not lead to an increase of R-squared (i.e., coefficient of determination) [43–45].

### Extreme bound analysis method

Although developing a predictive model using the regression method helps researchers investigate relationships among dependent and influential variables; the results might not be clear. In other words, a predictor variable that would be significant in one specification might not be insignificant in another. Therefore, the obtained results might depend on the selection of independent variables, which often vary across different studies. Thus, the EBA method was proposed by Leamer [46] and Leamer and Leonard [47] to address the uncertainty by measuring the sensitivity of regression estimates and answering the question: “Which influential predictors are robustly connected with the dependent variable?” In Leamer’s view, a predictor is robust if and only if its statistical significance is not conditional on the information set, that is, on whether other variables are added to and/or excluded from the regression equation. More specifically, the EBA method assists in measuring which of the influential predictors in the developed predictive model are robust and which of them are not robust and fragile. In fact, implementing this method helps determine the most extreme possible estimates, measure specification uncertainty, and examine the coefficients’ fragility.

Levine and Renelt [48] and Sala-i-Martin [49] implemented EBA to perform sensitivity and robustness analyses of influential predictors. Chanegriha et al. [50] adopted the EBA method, and their output supported the idea that EBA helps determine the robustness of the influential predictors. Chanegriha et al. [51] conducted a study on the implementation of the EBA method and determined that EBA changes the set of variables used for controlling and consequently determines the widest range coefficient estimates not rejected. They also indicated that the influential predictors are robust as long as the estimated coefficients are statistically significant.

The EBA method begins with running regression models, each consisting of a set of exploratory variables (***F***), a dependent variable (*y*), and a set (***D***) of the variables in ***X***. Accordingly, ***X*** and ***F*** refer to doubtful variables and free variables, respectively. The doubtful variables (i.e., ***X***) are labeled robust, while the others are considered fragile. The regression models can be estimated by using following equation:

$$y=\alpha_{i}+\beta_{i}v+\gamma_{i}F+\delta_{i}D_{i}+\varepsilon$$ (4)

where *i* indicates the generated models, ***F*** shows a set of exploratory variables, ***D***_***i***_ refers to a *k-*variables vector from ***X***, *ε* is the error, and β_i_ is the estimated coefficients of the focus variable (*v*).

**Fig 2:**
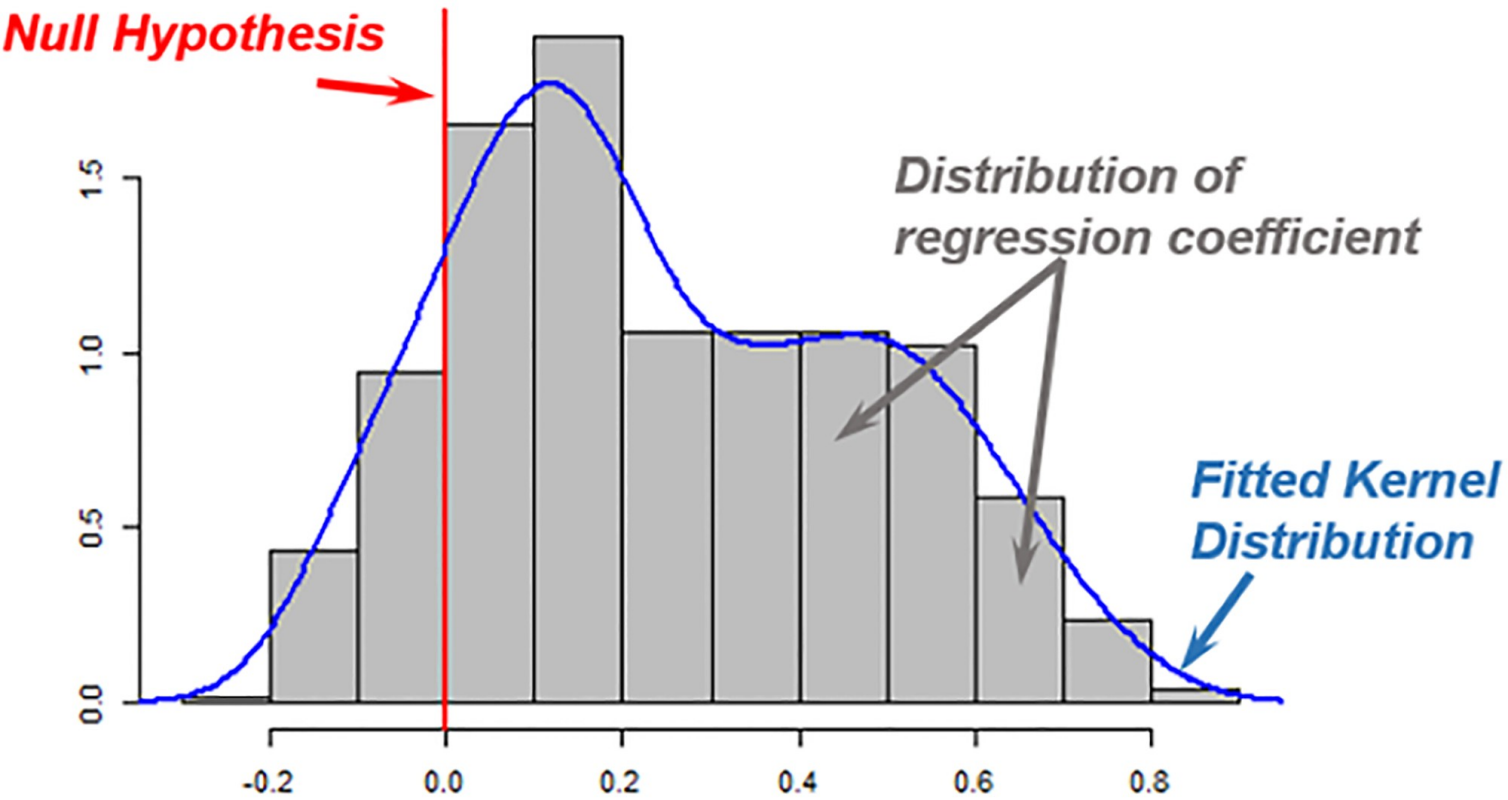
Illustration of EBA null hypothesis.

In the EBA version developed by Leamer, the lower and upper bounds of the regression coefficients would be used to investigate whether each influential predictor is robust or fragile [38]. For each influential variable, *v*, the extreme bounds refer to the highest and lowest values of ${\hat{\beta }}_{i}\pm \alpha {\hat{\sigma }}_{i}$ across the *N* number of estimated regression models, and α refers to the confidence level. As presented in Fig 2, the null hypothesis was set at zero. When the lower and upper bounds have the same signs, an influential variable is labeled a robust predictor; otherwise, it is considered a fragile predictor. The EBA method proposed by Leamer [46] has a strict criterion because an influential variable would be considered fragile even if its lower and upper bounds have the same signs in all of the *N* number of estimated regression models except one [46, 48, 49].

Sala-i-Martin [49] developed another version of the EBA method that instead of focusing on just extreme bounds, it would focus on the entire distribution of regression coefficients. In this version of the EBA method, a predictor would be labeled robust if at least 95% of its coefficient estimate was located on the same side of zero. In the Sala-i-Martin version of EBA method coefficient estimates across the *N* number of estimated regression models are assumed to follow (1) a normal distribution; or (2) a generic distribution, which would be scattered in less certain patterns.

## Research results

### Descriptive data analysis

**Fig 3:**
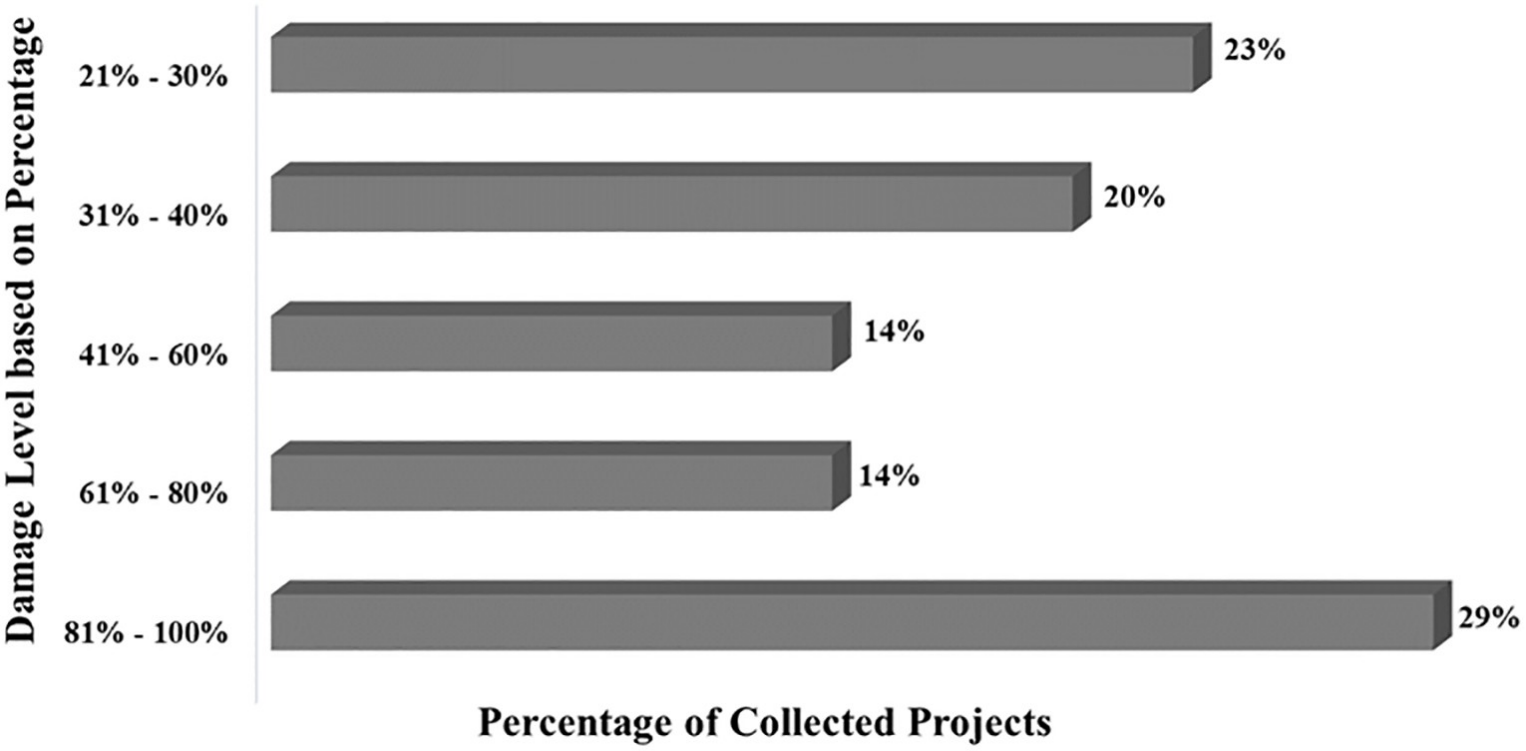
Damage level of transport infrastructure by hurricanes.

The respondents were asked to provide data on the damage level (i.e., compared to pre-disaster condition) sustained by the transportation infrastructure, and the results shown in Fig 3, indicate that in about 45% of the case studies, at least 60% of the entire structure was damaged, and in about 35%, 30 to 60% was damaged. The minimum damage level sustained by the whole structure of transport infrastructure was 21%.

The cost and schedule performance data from the 30 completed projects were analyzed descriptively, and the outcomes are presented in Table 4. Table 4 shows that the mean of the baseline budget and actual costs were approximately $25 million and $35 million, respectively; the mean of the actual time and baseline schedule were 18 months and 11 months, respectively.

**Table 4:** Descriptive data analysis.

Category		Minimum	Mean	Maximum	Standard Deviation
Cost	Baseline Budget	$0.3M	$23M	$100M	$33.2M
	Actual Cost	$0.5M	$36M	$150M	$53M
Schedule	Baseline Schedule	3 Months	11 Months	30 Months	9 Months
	Actual Time	6 Months	18 Months	42 Months	12 Months

The average values of variables with continuous data associated with two groups of good and poor cost performance were descriptively compared, and the results are shown in Table 5. It is worth mentioning that cost performance above 10% is labeled poor cost performance, and cost performance less than 10% is labeled a good cost performance [39].

**Table 5:** Comparison of variables with continuous data in reconstruction projects with good and poor cost performance.

Variables	Average	
	Poor Cost	Good Cost
	Performance	Performance
Number of main/truck lines	10	5
Total lengths of reconstruction	177.45 mi	37.85 mi
Distance from highly-populated area	34 mi	20 mi
Volume of debris	279166 CY**	51200 CY
Work suspension through execution of the project	1 month	1 week
Addressing environmental/safety issues prior to the project’s execution	3 months	1 week

*mi refers to mile;

**CY refers to cubic yard

Table 5 shows that the average volumes of debris for reconstruction projects with good and poor cost performance were approximately 50,000 CY and 280,000 CY, respectively. Table 5 also presents that the average lengths of reconstruction projects with poor and good cost performance were roughly 180 mi and 40 mi, respectively. The results in Table 5 demonstrate that the nature of reconstruction projects with good cost performance were less complicated than those with poor cost performance.

**Fig 4:**
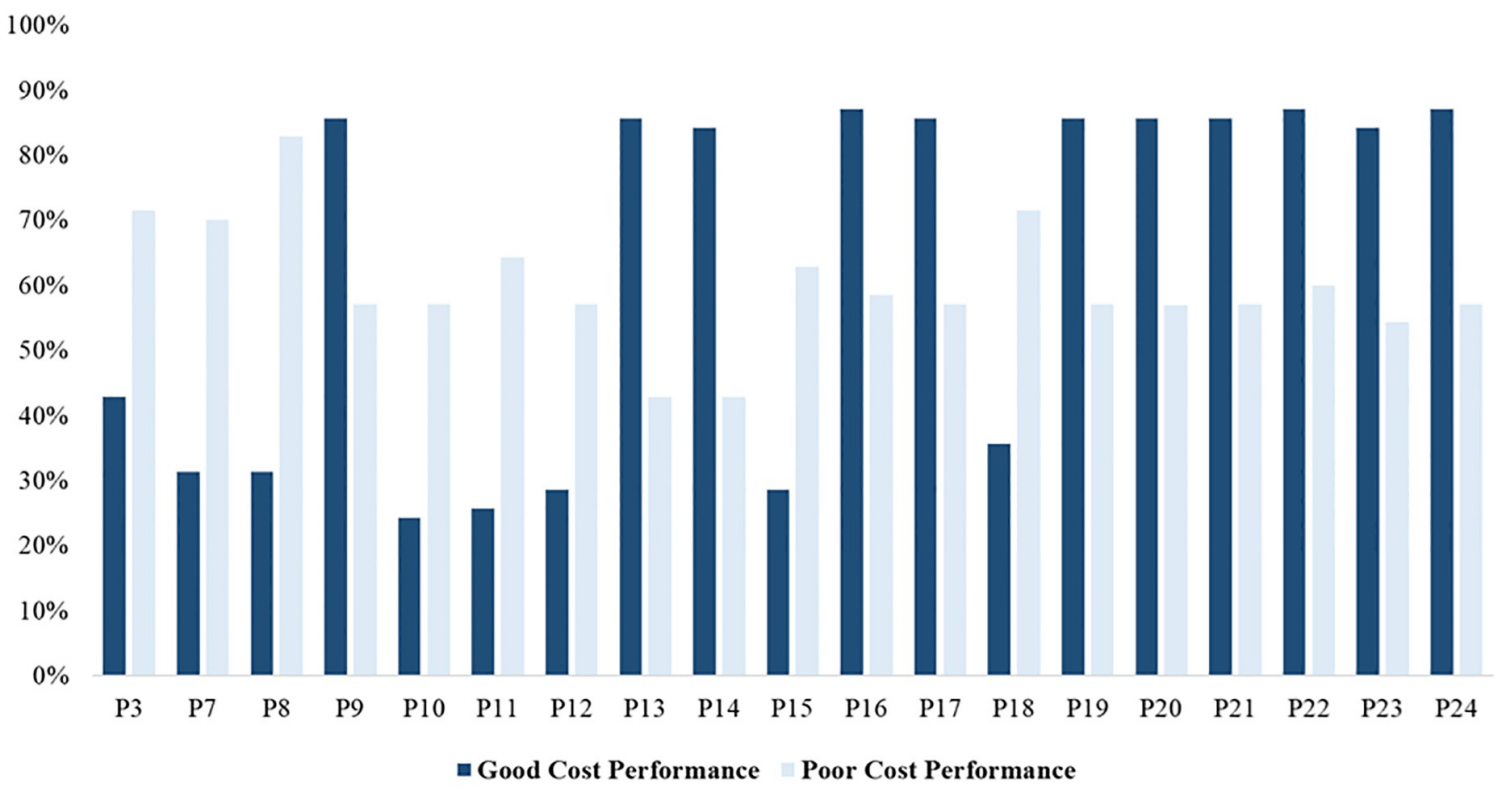
Comparison of scores of reconstruction cost performance predictors- Likert-scale data.

Furthermore, the mean values of good and poor cost performance associated with seven-point Likert-Scale responses were comparatively analyzed, and the results are shown in Fig 4. The mean values of the Likert-scale data were presented based on the percentage values to make them understandable and readable.

Fig 4 shows that the mean values of poor and good cost performance differed significantly. As indicated in Fig 4, the mean values of shortage of materials (P10) and shortage of equipment (P11) in projects with poor cost performance were significantly higher than those with good cost performance. Fig 4 also presents that the mean values of effective risk management (P23) and coordination (P24) in projects with good cost performance are significantly higher than those with poor cost performance.

The mean values of variables with continuous data associated with two groups of poor and good schedule performance were descriptively compared, and the results are shown in Table 6. It is worth mentioning that poor schedule performance is considered more than 20%, and schedule performance less than 20% is considered a good schedule performance [39].

**Table 6:** Comparison of variables with continuous data in reconstruction projects with good and poor schedule performance.

Variables	Average	
	Poor Schedule	Good Schedule
	Performance	Performance
Number of truck/main lines	11	4
Distance from highly-populated area	32 mi	16 mi
Addressing environmental/safety issues prior to the project’s execution	3.5 months	2 weeks
Work suspension through execution of the project	1.5 months	2 weeks

Table 6 illustrates that there were significant differences between the average values of the two groups of reconstruction projects with good schedule performance and poor schedule performance. For instance, as presented in Table 6, the average distances between the projects’ locations and highly-populated areas in projects with poor schedule performance and good schedule performance were 32 miles and 16 miles, respectively.

**Fig 5:**
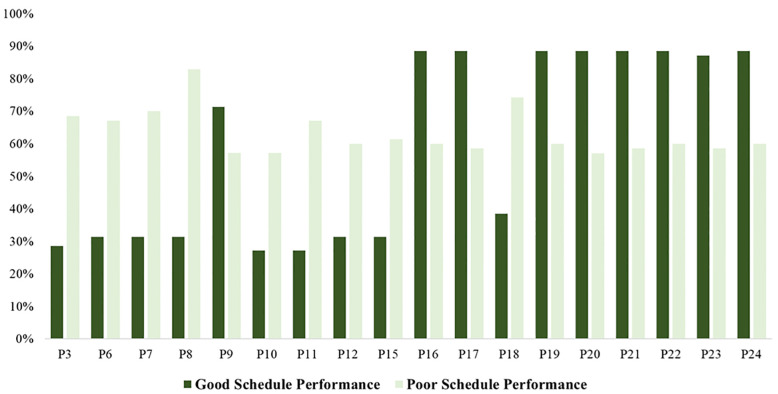
Comparison of scores of reconstruction schedule performance predictors- Likert-scale data.

Fig 5 presents the average scores’ differences between two groups of reconstruction projects with poor and good schedule performance based on the collected seven-point Likert scale responses. Similar to Fig 5, the average scores of the Likert-scale data were converted and presented based on percentages to improve the understandability and readability of the results.

Fig 5 presents that the average scores of variables in reconstruction projects with poor schedule performance were significantly different from those with good schedule performance. As shown in Fig 5, the average scores of shortage of materials (P10) and shortage of equipment (P11) in reconstruction projects with good schedule performance were considerably lower than those with poor schedule performance. In addition, the adoption level of risk management (P23) and coordination (P24) in projects with poor schedule performance was significantly lower than in reconstruction projects with good schedule performance.

### Development of a regression model for cost performance in reconstruction projects

As mentioned earlier, to collect comprehensive data and information on 30 case studies, a structured survey was designed, distributed, and collected 30 completed surveys associated with the same 30 case studies. Then, the reliability test using Cronbach’s alpha was performed and the calculated value obtained 0.79 indicating that the survey has adequate internal reliability.

The results of three statistical analysis including two-sample *t*-test (for numerical responses), Chi-square (for binary responses), and Kruskal-Wallis (Likert-scale responses) demonstrated that 26 predictors were determined statistically significant for cost performance in the post-hurricane reconstruction of transportation infrastructure.

The z-transformation method was adopted for both the dependent and independent variables to normalize and standardize the collected data. It is important to mention that for the binary (yes and no) responses, the values were 1 for “yes” and 0 for “no” responses. After auto-scaling the data, bidirectional stepwise multiple linear regression was used to predict the reconstruction cost performance in transportation infrastructure damaged by hurricanes. For this purpose, 26 determined significant predictors were used to develop the predictive model. Table 7 shows that the generated cost performance regression received an Adjusted *R*-Squared of 0.924 (92.4%), indicating that 92.4% of the cost performance is described by the regression equation.

**Table 7:** Results of stepwise multiple linear regression for cost performance in reconstruction projects.

Model Parameter	Value
R	0.976
R^2^	0.953
Adjusted R^2^	0.924
Standard Error	0.2012
Significance	0.000
*F*-Value	32.215
Durbin-Watson	1.639

The seven variables obtained as significant predictors in the model for cost performance are presented in Table 8: (1) increases in labor wages (P12), (2) accommodation level for the workforce (P14), (3) number of logistics management problems (P18), (4) number of inspections (P20), (5) information and knowledge management (P21), (6) pace of decision-making process (P22), and (7) addressing environment/safety problems prior to the project’s execution (P27).

**Table 8:** Summary of model variables for cost performance in reconstruction projects.

Independent Variables	Coefficient B	Standard Error	Beta	T	*P*-Value
Constant	0.106	0.007	-	14.174	0.000
P12	-0.061	0.008	-0.524	-7.350	0.000
P22	-0.046	0.011	-0.588	-4.354	0.001
P27	-0.068	0.010	-0.505	-6.785	0.000
P20	-0.026	0.006	-0.306	-4.545	0.001
P18	0.061	0.010	0.472	5.893	0.000
P14	0.032	0.013	0.306	2.369	0.037
P21	0.017	0.007	0.185	2.280	0.044

Table 8 shows the results of the unstandardized (B) and standardized (Beta) coefficients associated with the significant predictors, including the standard errors from the coefficients and the levels of significance of the cost performance predictors. The regression equation for reconstruction cost performance is expressed as follows:

$$\text{Reconstruction}\;\text{Cost}\;\text{Performance}=0.106+(-0.524)*\left(\text{P}12\right)+(-0.588)*\left(\text{P}22\right)+\left(-0.505\right)*\left(\text{P}27\right)+\left(-0.306\right)*\left(\text{P}20\right)+\left(0.472\right)*\left(\text{P}18\right)+\left(0.306\right)*\left(\text{P}14\right)+\left(0.185\right)*\left(\text{P}21\right)$$ (5)

where P12 refers to increases in labor wages, P14 refers to on-site accommodation level for staff, P18 refers to the frequency level of logistics management issues, P20 refers to the frequency of on-site inspection, P21 refers to information management, P22 refers to the pace of the decision-making process, and P27 refers to addressing environmental/safety issues prior to the project’s execution.

Table 8 illustrates that information management (P21) significantly enhances the cost performance in reconstruction projects. A successful data management system disseminates important information and data to all of the involved parties in a timely manner, thus minimizing the frequency of errors and mistakes and reducing overruns. The number of times that inspections are conducted (P20) helps project managers recognize issues and detect reconstruction risks so that they can be evaluated and mitigated or eliminated. They also enable authorities and decision-makers to verify that the standards and codes have been implemented appropriately, thus enhancing the cost performance of the projects.

Furthermore, logistic management (P18) impacts the cost of post-hurricane reconstruction projects. Damages sustained by transportation infrastructure result in delays in the delivery of machinery and materials needed for the reconstruction efforts, as many of the direct routes are not available and transportation facilities are not usually adequate to transfer the resources. Therefore, addressing these challenges increases the cost overruns in reconstruction projects and negatively affects the cost performance.

The homogeneity of variances was verified using an analysis of residuals to validate the developed predictive model. The outcomes showed that the residuals did not follow a certain pattern and behaved randomly, demonstrating that the assumption of homogeneity of variance was met and the model fitted the data well.

### Development of a regression model for schedule performance in reconstruction projects

Since the survey was designed to collect three types of responses, continuous value, Seven-point Likert-scale, and binary responses, three statistical analysis methods were adopted to determine statistically significant cost performance predictors. The two-sample *t*-test was implemented for continuous responses; Chi-square and Kruskal-Wallis methods were adopted for binary and Likert-scale responses, respectively. The results demonstrated that 23 predictors were determined statistically significant for schedule performance in the post-hurricane reconstruction of transportation infrastructure.

The method of z-transformation was adopted to normalize the data for both the dependent and independent variables. As mentioned earlier, for the binary (yes and no) responses, the values were 1 for “yes” and 0 for “no” responses. After standardizing the data, bidirectional stepwise multiple linear regression was used to predict the post-hurricane reconstruction schedule performance of transportation infrastructure. To this end, 23 determined significant predictors were used to develop the predictive model. Table 9 depicts the results and shows that the regression equation for reconstruction schedule performance, based on 30 observations, received an Adjusted *R*-Squared of 0.992 (99.2%). In this case, the value of Adjusted *R*-Squared demonstrates that 99.2% of the schedule performance is described by the generated regression equation.

**Table 9:** Results of stepwise multiple linear regression for schedule performance in reconstruction projects.

Model Parameter	Value
R	0.998
R^2^	0.996
Adjusted R^2^	0.992
Standard Error	0.00395
Significance	0.000
*F*-Value	265.088
Durbin-Watson	1.738

Nine significant predictors of schedule performance were identified by implementing the stepwise multiple regression procedure: (1) level of project complexity (P3), (2) level of traffic disturbances (P6), (3) shortage of materials (P10), (4) shortage of equipment (P11), (5) increases in labor wages (P12), (6) shortage of suppliers (P12), (7) number of inspections (P20), (8) data management (P21), and (9) addressing the environment/safety issues prior the project’s execution (P27). The homogeneity of variances was evaluated by analyzing the residuals to validate the schedule performance regression model, and the result indicated that the residuals completely behave randomly.

Table 10 depicts the outcomes of both the standardized (Beta) and unstandardized coefficients (B) associated with the significant predictors. The regression equation (Eq 6) for the reconstruction schedule performance is expressed as follows:

$$\text{Reconstruction}\;\text{Schedule}\;\text{Performance}=0.099+\left(-0.606\right)*\left(\text{P}12\right)+\left(-0.844\right)*\left(\text{P}20\right)+\left(-0.600\right)*\left(\text{P}6\right)+\left(-0.547\right)*\left(\text{P}27\right)+\left(0.193\right)*\left(\text{P}3\right)+\left(0.375\right)*\left(\text{P}15\right)+\left(0.281\right)*\left(\text{P}11\right)+\left(0.159\right)*\left(\text{P}10\right)+\left(-0.100\right)*\left(\text{P}21\right)$$ (6)

where P3 refers to complexity level, P6 refers to traffic disturbance level, P10 refers to shortage of materials, P11 refers to shortage of equipment, P12 refers to increases in labor wages, P15 refers to shortage of supplier, P20 refers to frequency of on-site inspection, P21 refers to information management, P27 refers to addressing environmental/safety issues prior to the project’s execution.

**Table 10:** Summary of model variables for schedule performance in reconstruction projects.

Independent Variables	Coefficient B	Standard Error	Beta	T	*P*-Value
Constant	0.099	0.001	-	68.369	0.000
P12	-0.050	0.004	-0.606	-11.647	0.000
P20	-0.063	0.003	-0.844	-23.311	0.000
P6	-0.045	0.003	-0.600	-13.377	0.000
P27	-0.046	0.003	-0.547	-13.965	0.000
P3	0.017	0.002	0.193	7.000	0.000
P15	0.031	0.003	0.375	9.527	0.000
P11	0.025	0.004	0.281	5.651	0.000
P10	0.012	0.003	0.159	4.309	0.002
P21	-0.009	0.003	-0.100	-3.303	0.009

Table 10 shows that reconstruction projects for damaged transport infrastructure have become increasingly complicated (P3) and require experienced, skilled, and knowledgeable project management teams and craft laborers to execute the projects with a minimum of errors. In fact, since this type of workforce is often unavailable after disasters, this seriously affects the projects’ schedule performance and consequently, the project complexity increases. Table 10 also indicates that heavy traffic and traffic disturbances (P6) are also a predictor of schedule performance. This is justifiable because heavy traffic and bigger traffic disturbances cause the efficiency and safety of traveling to reduce through the work zones on the one hand, and on the other hand lead to increase in the number of accidents and prevent the timely delivery of materials and equipment.

The shortages of materials (P10) and shortages of equipment (P11) shown in Table 10 were recorded as two predictors that contribute to time delays in post-hurricane reconstruction and negatively impact the schedule performance. Table 10 also shows that increases in labor wages (P12) lead to more satisfied and motivated laborers, which results in increased productivity [49] and improved schedule performance. Table 10 indicates that addressing environmental and safety issues prior to a project’s execution (P27) decreases the probability of serious problems occurring in those areas and keeps the schedule on track.

The homogeneity of variances was verified using an analysis of residuals to validate the developed model to predict reconstruction schedule performance. The outcome demonstrates that the residuals did not follow a specific pattern and behaved randomly, indicating that the model fitted the data well and the assumption of homogeneity of variances was met.

### Extreme bound analysis of cost performance predictors

The form of the EBA method suggested by [49] was implemented to determine the robust or fragile connection between each cost performance predictor and the generated regression model, and the results are presented in Table 11. The four robust predictors for cost performance are: (1) increases in labor wages (P12), (2) quantity and quality of logistics management problems (P18), (3) number of inspection times (P20), and (4) information and data management (P21).

**Table 11:** Results of EBA analysis for cost performance predictors.

Predictor	Sala-i-Martin’s EBA Result				Type of Predictor
	Normal CDF (*β* ≤ 0)	Normal CDF (*β* > 0)	Non-Normal CDF (*β* ≤ 0)	Non-Normal CDF (*β* > 0)	
Intercept	30.705	69.295	33.344	66.656	Fragile
P12	0.549	99.451	1.342	98.658	Robust
P14	0.223	99.777	7.053	92.947	Fragile
P18	6.965	93.035	9.517	95.483	Robust
P20	5.241	94.759	6.901	98.099	Robust
P21	0.000	100.000	0.000	100.000	Robust
P22	17.519	82.481	22.884	77.116	Fragile
P27	17.786	82.214	21.614	78.386	Fragile

Table 11 shows that logistics management issues (P18) significantly affect post-hurricane reconstruction costs and are also a robust predictor (Table 11). One example is that without effective logistics management, shipping costs are higher because orders are rushed and/or are shipped in inappropriate transporting materials, which increases cost overrun values.

The frequency of on-site inspections (P20) was also obtained as a robust predictor for the cost performance of the targeted projects (Table 11). On-site inspections play a critical role in preventing serious issues because inspectors would identify the potential risks at the right time and project managers would conduct proactive plans. An inadequate inspection usually causes a need for additional funding to address problems as they arise.

**Fig 6:**
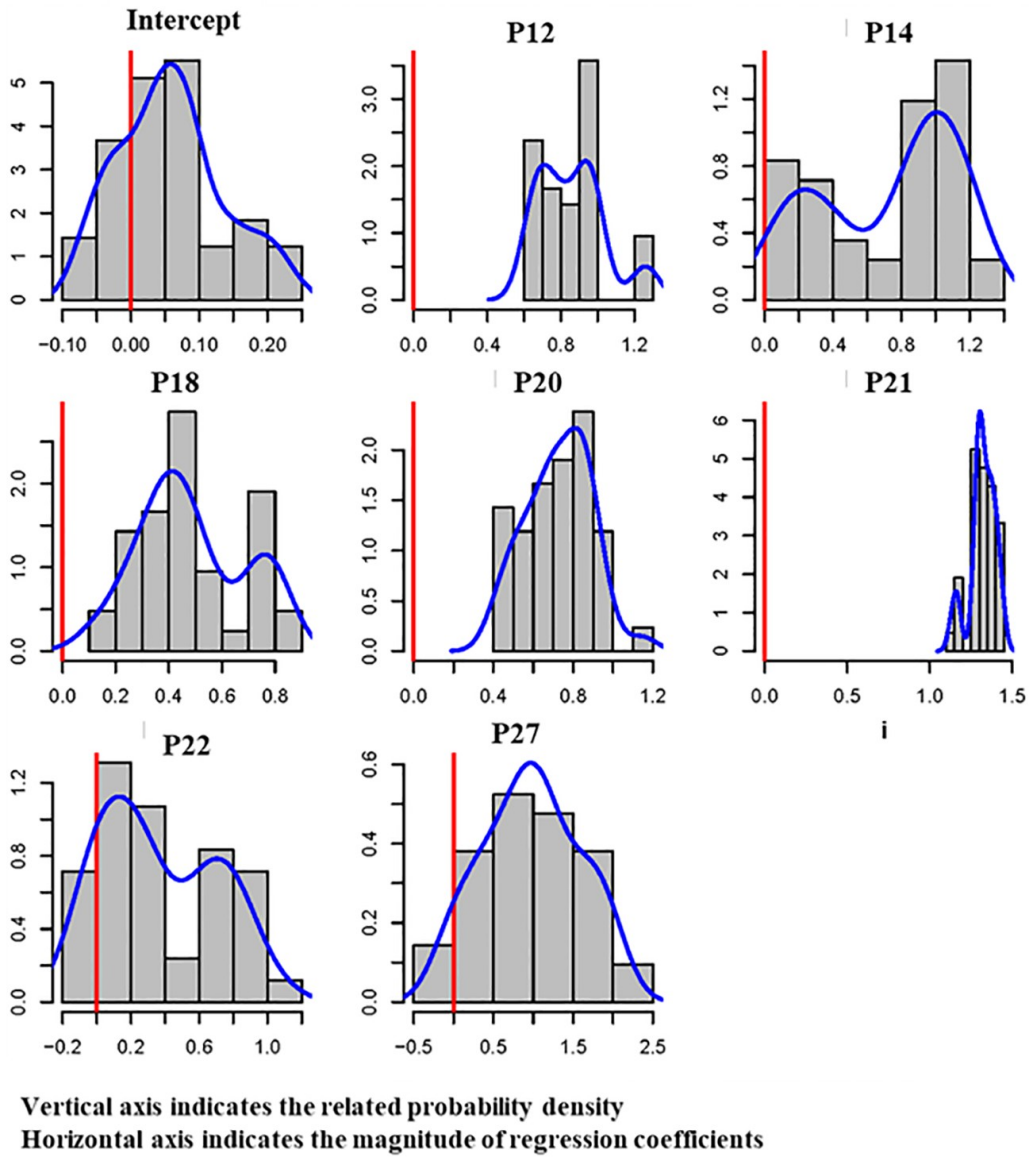
Schematic results of EBA for reconstruction cost performance predictors.

Information management (P21) was also recorded as a robust predictor for the cost performance of reconstruction projects. Lack of effective information management results in information and data not being shared in a timely manner among the stakeholders and project parties, thereby seriously increasing the number and extent of risks to strategic plans, adding to the cost and decreasing the cost performance. Fig 6 schematically presents the results of the EBA method for reconstruction cost performance and shows that four predictors (Ps 12, 18, 20, and 21) are robustly connected to the regression equation.

### Extreme bound analysis of schedule performance predictors

The EBA method was conducted to investigate and determine which schedule performance predictors were robustly connected to the developed predictive model. The EBA method suggested by [38] was conducted, and the outcomes are illustrated in Table 12, which shows that six predictors are robust for the schedule performance of post-disaster reconstruction projects: (1) level of complexity (P3), (2) level of traffic disturbances (P6), (3) shortage of materials (P10), (4) shortage of equipment (P11), (5) shortage of suppliers (P15), and (6) frequency of on-site inspections (P20).

**Table 12:** Results of EBA analysis for schedule performance predictors.

Predictor	Sala-i-Martin’s EBA Result				Type of Predictor
	Normal CDF (*β* ≤ 0)	Normal CDF (*β* > 0)	Non-Normal CDF (*β* ≤ 0)	Non-Normal CDF (*β* > 0)	
Intercept	20.831	79.169	24.487	75.513	Fragile
P3	0.025	99.975	0.499	99.501	Robust
P6	0.000	100.000	0.001	99.999	Robust
P10	0.005	99.995	0.535	99.465	Robust
P11	0.604	99.396	3.439	96.561	Robust
P12	71.083	28.917	62.761	37.239	Fragile
P15	0.204	99.796	2.231	97.769	Robust
P20	0.229	99.771	2.209	97.791	Robust
P21	48.483	51.517	46.348	53.652	Fragile
P27	11.442	88.558	22.986	77.014	Fragile

As presented in Table 12, the complexity level of post-disaster reconstruction projects is inherently high (P3), which usually increases the number of challenges and issues encountered. Unfortunately, after hurricanes, at a time when skilled and experienced project managers and laborers are needed, they are frequently in short supply, which causes an increase in the number of errors and delays in the project. Table 12 demonstrates that traffic congestion and disturbance (P6) as a predictor is robustly connected to the developed predictive model because they do not allow timely delivering equipment and materials; so, the probability of time delays would significantly increase.

Shortages of materials (P10) and shortages of equipment (P11) are also robust predictors, and the availability of sufficient supply of both substantially leads to the timely reconstruction of the damaged transportation infrastructure. A shortage of suppliers (P15) after a disaster requires that different strategies be implemented to compensate for delayed deliveries or unavailability of materials and machinery. The time it takes to devise and execute new plans causes delays in the completion schedule. The frequency of on-site inspections (P20) is a robust predictor, as presented in Table 12. Risks are expected in large-scale reconstruction projects because of the chaotic and dynamic environment following hurricanes. The frequent on-site inspections allow early identifying serious issues and problems and taking steps to mitigate or prevent the damage they may cause. Therefore, this predictor would significantly improve the schedule performance of reconstruction projects.

**Fig 7:**
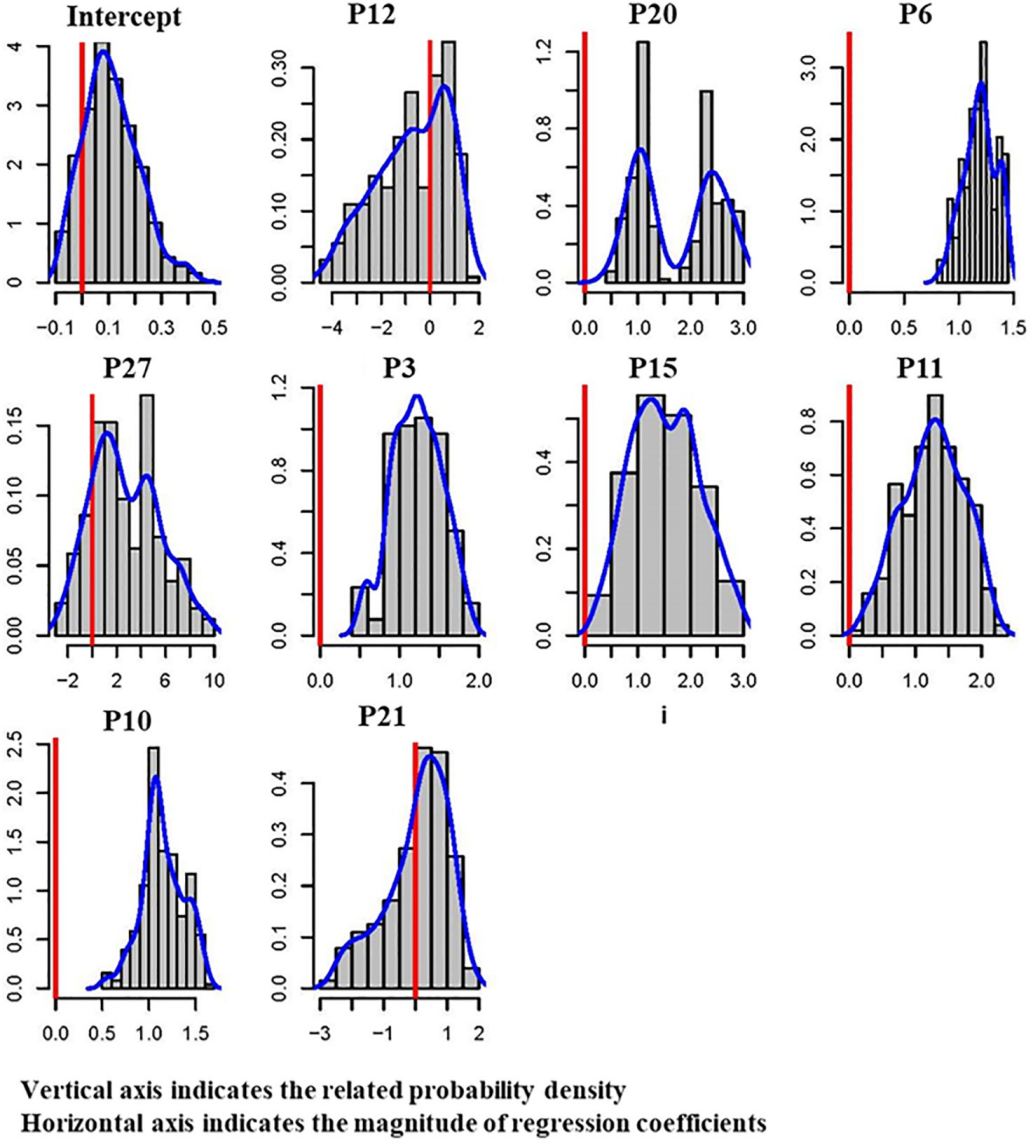
Schematic results of EBA for reconstruction schedule performance predictors.

Fig 7 presents the outcomes of EBA analysis schematically and illustrates those six predictors (P3, P6, P10, P11, P15, and P20) were robustly connected to the generated regression equation that predicted the reconstruction schedule performance.

### Comparative analysis of predictors

**Fig 8:**
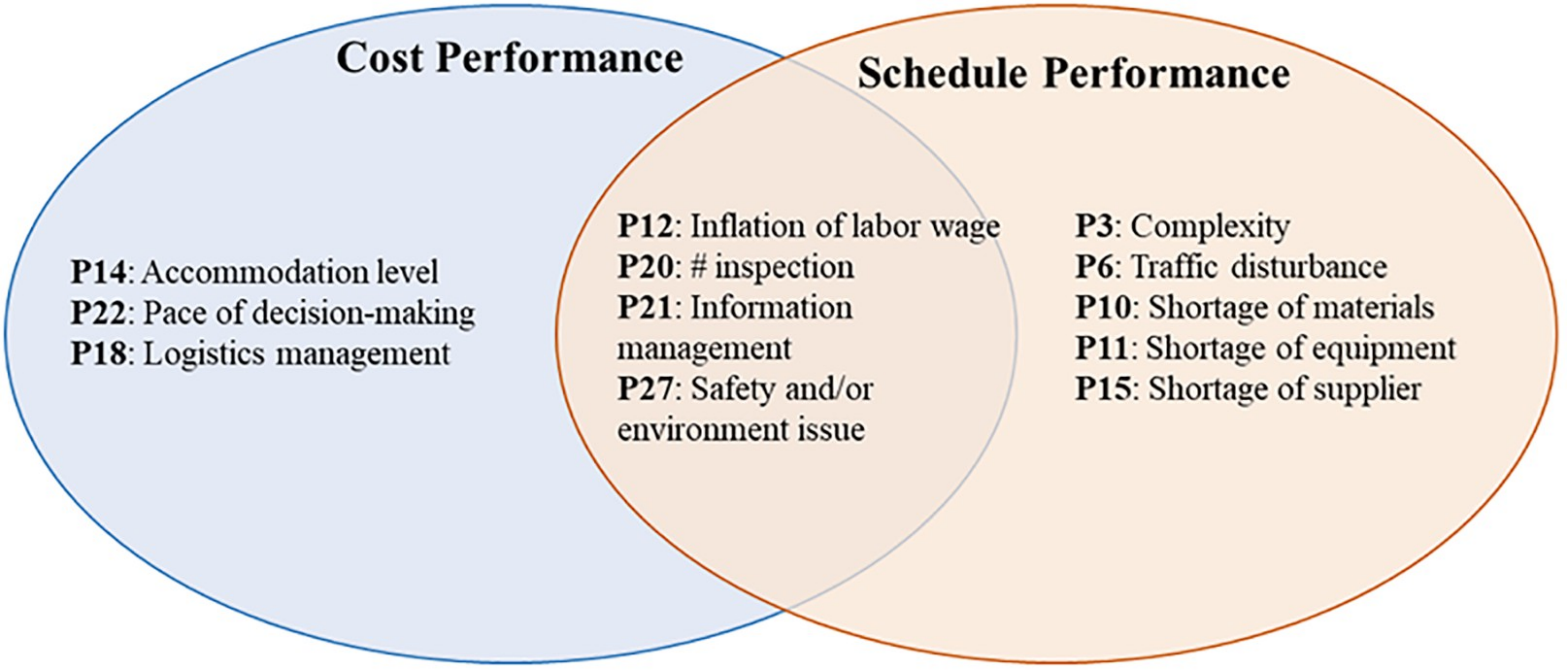
Comparative analysis of cost and schedule performance predictors.

A simple comparative analysis of cost and schedule performance predictors was conducted to determine which of the predictors are common between cost and schedule performance. These predictors include increases in labor wages (P12), number of inspections (P20), data management (P21), and addressing the safety/environment issues prior to the project’s execution (P27), as schematically presented in Fig 8.

Frequent on-site inspections (P20) are one of the shared predictors that substantially helps project management teams identify current and potential problems before they become major issues, thus mitigating, or preventing costly delays and overruns. Fig 8 shows that information management (P21) is another shared predictor that contribute to both the cost and schedule performances. Effective information management keeps the stakeholders and other project parties up to date on everything that affects the project’s success so that they can be proactive in preventing or at least mitigating any problems that arise that could decrease the cost and schedule performance.

### Comparative analysis of robust predictors

**Fig 9:**
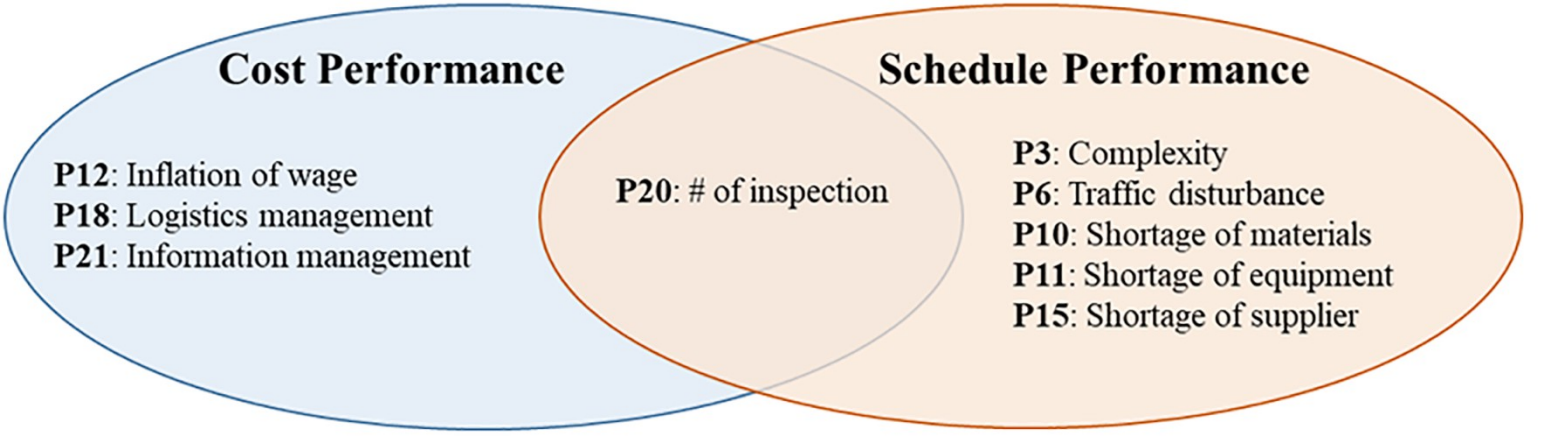
Comparative analysis of robust cost and schedule performance predictors.

A simple comparative analysis was conducted of the robust predictors that impact the time and cost of reconstruction projects in the aftermath of hurricanes. The results are presented in Fig 9, which indicates that the number of inspections (P20) is a robust predictor shared by both the cost and schedule performance. Inspections allow the quality of work and the project’s safety to be closely monitored so that serious challenges, cost overruns, and delayed completion of the project can be prevented.

## Model implementation

Project managers and decision-makers will be able to use the outcomes of this study to timely identify the critical predictors contributing to serious cost overruns and schedule delays, and make proactive plans to address predictors. Seven robust predictors of cost performance and nine robust predictors of schedule performance were revealed by this research. Furthermore, project managers and decision-makers will use the generated predictive models and equations to estimate cost and schedule performance of their post-hurricane reconstruction of transportation infrastructure.

Shortages of resources are common after hurricanes, and a support system is needed to assist project managers and decision-makers in allocating the available resources effectively. Implementing the EBA method allows practitioners to determine the most important factors for cost-effective recovery and reconstruction. Therefore, the outcomes of the EBA method assist project managers in prioritizing resource allocation from robust to fragile predictors that lead to minimizing cost escalations and delays in reconstruction projects.

Two case studies were used to validate the cost and schedule performance models. The data was collected by asking the employees involved with them to answer survey questions; their responses were utilized to develop the model. Table 13 provides general information about the case studies.

**Table 13:** Data from two case studies.

Characteristic	Project 1	Project 2
Baseline budget	$3M	$15M
Actual cost	$3.2M	$17M
Baseline schedule	2 years	2 years
Actual time	2.5 years	2.5 years
Cost performance	6%	13%
Schedule performance	25%	25%

Two regression equations were generated to predict the cost and schedule performances according to the collected data associated with the two case studies. Table 13 shows that Project 1 was completed with actual cost and schedule performances of 6% and 25%, respectively. Project 2 was completed with an actual cost performance of 13% and schedule performance of 25%. The predicted schedule performance for projects 1 and 2 are 23% and 22%, respectively, which are 2% and 3% less than the schedule performance of the completed projects. The predicted cost performance for projects 1 and 2 are 5% and 11%, respectively, which are 1% and 2% less than the actual cost performances of the projects. The estimated cost performance for projects 1 and 2 are 7% and 26% respectively, which are 3% and 4% less than the real cost performances of the projects. According to the comparison of the estimated cost and schedule performance of the two case study projects with the actual output of the projects, the model output is verified and close enough to the actual cost and schedule performance of both projects.

## Conclusions

Two predictive models were generated using stepwise multiple regression to estimate the cost and schedule performance of reconstructing transport infrastructure damaged by hurricanes. The study further determined which predictors are robustly connected to the developed models. It was concluded that seven cost performance predictors and nine schedule performance predictors accounted for Adjusted R-Squared of 92.4% and 99.2%, respectively. Four cost and seven performance predictors were robustly related to their corresponding cost and schedule performance predictive model. The three predictors in both developed models were the frequency of on-site inspections, information management, and addressing safety/environment issues prior to the project’s execution. The frequency of on-site inspections was a robust predictor shared by both the cost and schedule performance, as this robust predictor improves the quality and safety of the work by closely monitoring it so that serious issues and the corresponding time and cost can be prevented. The outcomes of this study will provide information to project managers and decision-makers to allocate limited resources effectively and substantially minimize cost escalations and time delays in the post-hurricane reconstruction of transportation infrastructure.

Although the research team of this study put substantial efforts into achieving valid and reliable results, this study contains a few shortcomings. First, the list of influential factors and potential predictors was identified through a review of the existing literature, but there might be some other critical factors that are applicable in the construction industry and not easy to find in the literature. This study also focused on the geographical and environmental context of the U.S. In addition, future research could expand this study by incorporating the chaotic environments into the models, as this would help decision-makers investigate the interactions and dynamics among influential factors throughout the execution of the project.

## Supporting information

S1 File Appendix. (PDF)
