# Supplementary material for: Prediction of cost and schedule performance in post-hurricane reconstruction of transportation infrastructure
Source: PLoS One. 2023 Mar 29;18(3):e0282231. doi: 10.1371/journal.pone.0282231 (PMC10057790; doi:10.1371/journal.pone.0282231)
Supplement: S1 File — (PDF) [file pone.0282231.s001.pdf]

Appendix

Survey Questions

Survey Instruction

Dear Participant,

We are conducting a study to estimate the cost and timeline of post-disaster reconstruction of transportation infrastructures due to natural extreme events. Given your expertise in the field, we believe your input and feedback would be very valuable. The project would greatly benefit from your insights.

The survey requires about 10-15 minutes to complete. Your participation is voluntary and your responses to the survey will be kept strictly confidential.

If you have any questions, you may contact the project Principal Investigator, Dr. Sharareh (Sherri) Kermanshachi, at 817-272-2704 or sharareh.kermanshachi@uta.edu.

We appreciate your participation in advance.

Respondent Information

1. How many years of work experience do you have?

2. What is your current position in the agency/company you are working in?

- ○ Director
- ○ Project engineer
- ○ Field labor
- ○ Project manager
- ○ Other

Area Transportation Network

3. What type of disaster was the cause of damages to the selected reconstruction project?

- Hurricane
- Flood
- Thunderstorm
- Tornado
- Earthquake
- Tsunami
- Other

18

4. What year did the selected disaster happen?

5. In which state did the selected disaster happen?

Alabama
Alaska
Arizona
Arkansas
California
Colorado
Connecticut
Delaware
Florida
Georgia

19

6. Approximately, how many reconstruction projects of transportation infrastructures were needed in the affected area due to this disaster?

20

7. Approximately, how many of the reconstruction projects were completed by your company/agency in the selected affected area?

8. What was the role of your company in the selected reconstruction project?

- Owner
- Contractor
- Engineer/Designer
- Other

21

Project-Based Information

General Information

9. Approximately, what were baseline budget and actual cost of the selected reconstruction project due to this disaster?

Note: This question is very important for the research team. Please provide response to this question.

Baseline budget

Actual cost

10. Approximately, what were the baseline schedule and the actual time of the selected reconstruction project due to this disaster?

Note: This question is very important for the research team. Please provide response to this question.

22

Baseline schedule

Actual time

23

11. Approximately, what was the cost ($) of delay per day (Liquidated damage) in the selected reconstruction project?

12. What was total cost of the reworks (e.g. repairing defects) during the construction of the selected project?

24

Physical Characteristics of the Project

13. Approximately, how many main/trunk lines did the selected reconstruction project consist of? (Main/trunk line refers to the primary linkage serving main arteries of interaction and commerce in transportation networks)

14. Approximately, what was the length of the selected reconstruction project?

15. Please rate the complexity level of the selected reconstruction project.

Not at All Complex (1)	(2)	(3)	Fairly Complex (4)	(5)	(6)	Extremely Complex (7)
○	○	○	○	○	○	○

16. How remote (distance from highly-populated areas) was the project located?

Damaging Level

17. Approximately, what was level/percentage of damages in the selected reconstruction project compared to its pre-disaster condition?

	0%-10%	11%-20%	21%-30%	31%-40%	41%-60%	61%-80%	81%-100%
Damaging Level	☐	☐	☐	☐	☐	☐	☐

18. What level of traffic/transportation disturbance did your company experience through the construction of the selected project?

No Traffic			Light Traffic			Heavy Traffic
(1)	(2)	(3)	(4)	(5)	(6)	(7)

Resources

19. Please rate shortage of the needed experts in the selected reconstruction project.

No Shortage			Moderate Shortage			Severe Shortage
(1)	(2)	(3)	(4)	(5)	(6)	(7)

20. Please rate shortage of field labors in the selected reconstruction project.

No Shortage			Moderate Shortage			Severe Shortage
(1)	(2)	(3)	(4)	(5)	(6)	(7)

29

---

21. Please rate level of competency and/or productivity of contractors in the selected
30 reconstruction project.

Not at All Competent	(2)	(3)	Moderately Competent	(5)	(6)	Highly Competent
(1)			(4)			(7)

31

22. Please rate frequency level of logistics management issues in the selected reconstruction project. (Logistics management is the process of planning, implementing and controlling supply chain resources from the point of origin, such as raw material accumulation, to the correct location on the construction site.)

Limited No. of Issues			Reasonable No. of Issues			Substantial No. of Issues
(1)	(2)	(3)	(4)	(5)	(6)	(7)

32

23. Please rate shortage of competent suppliers in the selected reconstruction project.

			Moderate			Severe
No Shortage			Shortage			Shortage
(1)	(2)	(3)	(4)	(5)	(6)	(7)

24. Please rate shortage of materials in the selected reconstruction project.

			Moderate			Severe
No Shortage			Shortage			Shortage
(1)	(2)	(3)	(4)	(5)	(6)	(7)

25. Please rate shortage of equipment in the selected reconstruction project.

			Moderate			Severe
No Shortage			Shortage			Shortage
(1)	(2)	(3)	(4)	(5)	(6)	(7)

33

26. Please rate quality issues with materials in the selected reconstruction project.

			Moderate			
Poor Quality			Quality			High Quality
(1)	(2)	(3)	(4)	(5)	(6)	(7)

34

27. Please rate quality issues with equipment in the selected reconstruction project.

			Moderate			
Poor Quality			Quality			High Quality
(1)	(2)	(3)	(4)	(5)	(6)	(7)

28. What level of labor wage rate inflation (i.e. extend of demand surge) did market experience through construction of the selected reconstruction project?

			Moderate			
No Inflation			Inflation			High Inflation
(1)	(2)	(3)	(4)	(5)	(6)	(7)

35

29. What was the availability level of the existing infrastructures on-site to support the selected reconstruction project?

No Infrastructure			Limited Infrastructure			Available Infrastructure
(1)	(2)	(3)	(4)	(5)	(6)	(7)

30. What was the accommodation level for staff/labors on-site in the selected reconstruction project? (Accommodation refers to providing of what is needed for the convenient working environments such as availability of existing housing and medical services)

No Accommodation			Limited Accommodation			Excellent Accommodation
(1)	(2)	(3)	(4)	(5)	(6)	(7)

36

Environment & Safety

31. Approximately, what level of debris was initially removed from the site of the selected reconstruction project?

37

32. Did you/your agency have to address/settle any environmental issues (e.g. the act of dewatering) before starting the construction of the selected reconstruction project?

Yes

No

If your answer to the previous question is **Yes**, please answer the following question:

33. Approximately, what was the duration for settling/addressing the environmental issues before starting the execution of the selected reconstruction project?

38

34. Was construction of the selected project suspended due to intolerable weather conditions (e.g. extreme heat and cold weather)?

- ◯ Yes
- ◯ No

If your answer to the previous question is **Yes,** please answer the following question:

35. Approximately, what was the duration of work suspension through construction of the selected project?

[▼]

36. Did your company/agency limit the site accessibility due to safety concerns and/or other concerns through construction of the selected project?

- ◯ Yes

39 ◯ No

If your answer to the previous question is **Yes,** please answer the following question:

37. Approximately, what was the duration of site inaccessibility through construction of the

40 selected project?

[▼]

41

Project Management

42

37. Please rate quality level of on-site inspection in the selected reconstruction project.

Poor Quality (1)	(2)	(3)	Moderate Quality (4)	(5)	(6)	High Quality (7)
◯	◯	◯	◯	◯	◯	◯

43

38. Please rate frequency level of on-site inspection in the selected reconstruction project.

Rare			Moderate			Frequent
(1)	(2)	(3)	(4)	(5)	(6)	(7)

39. Please rate level of information management (i.e. database information) in the selected reconstruction management.

No Management			Moderate Management			Effective Management
(1)	(2)	(3)	(4)	(5)	(6)	(7)

40. Please rate pace of decision-making process through construction of the selected project.

Very Slow			Moderate Speed			Very Fast
(1)	(2)	(3)	(4)	(5)	(6)	(7)

44

41. Please rate implementation level of risk analysis and management in the selected project.

Not at All Implemented	(2)	(3)	Adequately Implemented	(5)	(6)	Highly Implemented

45

(1) (4) (7)

46

42. Please rate pace of workers' mobilization in the selected reconstruction project.

Very Slow			Moderate Speed			Very Fast
(1)	(2)	(3)	(4)	(5)	(6)	(7)

43. Please rate effectiveness level of coordination in the selected reconstruction project.

Poor Coordination			Moderately Efficient			Effective Coordination
(1)	(2)	(3)	(4)	(5)	(6)	(7)

47

Local

44. Please rate availability of required temporary pathways in the selected project.

Completely Unavailable (1)	(2)	(3)	Moderate Availability (4)	(5)	(6)	High Availablity (7)

Legal

46. Please rate the level of regulatory requirements in the selected reconstruction project. (Regulatory requirements refer to any legally enforceable requirement of any regulator)

Not at All (1)	(2)	(3)	Moderate (4)	(5)	(6)	Extremely High (7)
